# Implementing a Social Needs Screening and Referral Program Among Veterans: Assessing Circumstances & Offering Resources for Needs (ACORN)

**DOI:** 10.1007/s11606-023-08181-9

**Published:** 2023-05-10

**Authors:** Lauren E. Russell, Alicia J. Cohen, Steven Chrzas, Christopher W. Halladay, Meaghan A. Kennedy, Kathleen Mitchell, Ernest Moy, Lisa Soleymani Lehmann

**Affiliations:** 1 Office of Health Equity, Veterans Health Administration, Washington, DC USA; 2 VA Health Services Research & Development (HSR&D) Center of Innovation in Long Term Services and Supports (LTSS-COIN), VA Providence Healthcare System, Providence, RI USA; 3 Department of Family Medicine, Warren Alpert Medical School of Brown University, Providence, RI USA; 4 Department of Health Services, Policy, and Practice, Brown University School of Public Health, Providence, RI USA; 5 VA Connecticut Healthcare System, West Haven, CT USA; 6 New England Geriatric Research, Education, and Clinical Center, VA Bedford Healthcare System, Bedford, MA USA; 7 Department of Family Medicine, Boston University School of Medicine, Boston, MA USA; 8 Department of Community and Family Medicine, Geisel School of Medicine at Dartmouth, Hanover, NH USA; 9 Department of Medicine, Harvard Medical School, Boston, MA USA; 10 Department of Health Policy and Management, Harvard T. H. Chan School of Public Health, Boston, MA USA; 11 Brigham and Women’s Hospital, Boston, MA USA

**Keywords:** social risk factors, social needs, social determinants of health, veteran, screening

## Abstract

**Background:**

The Department of Veterans Affairs (VA) healthcare system routinely screens Veterans for food insecurity, housing instability, and intimate partner violence, but does not systematically screen for other health-related social needs (HRSNs).

**Objectives:**

To (1) develop a process for systematically identifying and addressing Veterans’ HRSNs, (2) determine reported prevalence of HRSNs, and (3) assess the acceptability of HRSN screening among Veterans.

**Design:**

“Assessing Circumstances and Offering Resources for Needs” (ACORN) is a Veteran-tailored HRSN screening and referral quality improvement initiative. Veterans were screened via electronic tablet for nine HRSNs (food, housing, utilities, transportation, legal needs, social isolation, interpersonal violence, employment, and education) and provided geographically tailored resource guides for identified needs. Two-week follow-up interviews with a purposive sample of Veterans explored screening experiences.

**Participants:**

Convenience sample of Veterans presenting for primary care at a VA urban women’s health clinic and suburban community-based outpatient clinic (October 2019–May 2020).

**Main Measures:**

Primary outcomes included prevalence of HRSNs, Veteran-reported acceptability of screening, and use of resources guides. Data were analyzed using descriptive statistics, chi-square tests, and rapid qualitative analysis.

**Key Results:**

Of 268 Veterans screened, 50% reported one or more HRSNs. Social isolation was endorsed most frequently (29%), followed by educational needs (19%), interpersonal violence (12%), housing instability (9%), and utility concerns (7%). One in five Veterans reported at least one form of material hardship. In follow-up interviews (*n* = 15), Veterans found screening acceptable and felt VA should continue screening. No Veterans interviewed had contacted recommended resources at two-week follow-up, although several planned to use resource guides in the future.

**Conclusion:**

In a VA HRSN screening and referral program, Veterans frequently reported HRSNs, felt screening was important, and thought VA should continue to screen for these needs. Screening for HRSNs is a critical step towards connecting patients with services, identifying gaps in service delivery, and informing future resource allocation.

**Supplementary Information:**

The online version contains supplementary material available at 10.1007/s11606-023-08181-9.

## INTRODUCTION

With increasing recognition of the impact of social and behavioral determinants on health outcomes, policy makers, payers, and professional organizations have called for health systems to improve their means of identifying and addressing health-related social needs (HRSNs) including food insecurity, housing instability, interpersonal violence, and lack of transportation.^1–5^ While screening for HRSNs is increasingly being implemented in healthcare settings, widespread implementation remains relatively low.^6^

Veterans receiving care within the Department of Veterans Affairs (VA) healthcare system are routinely screened for housing instability^7^, food insecurity^8^, and intimate partner violence.^9^ However, VA does not systematically screen for HRSNs more broadly. To address this gap, our interprofessional team developed a Veteran-tailored HRSN screening and referral quality improvement initiative, “Assessing Circumstances and Offering Resources for Needs” (ACORN). ACORN aims to equip clinical teams with up-to-date information to better understand and address the social and economic factors impacting patients’ health.

We evaluated ACORN at 2 VA outpatient primary care clinics. Specifically, we assessed (1) prevalence of identified HRSNs; (2) Veteran-reported acceptability of ACORN; and (3) use of geographically tailored resource guides.

## METHODS

### Setting

ACORN was implemented at 2 VA New England Healthcare System primary care clinics: an urban women’s health clinic and a suburban community-based outpatient clinic. The clinics were selected based on strong local leadership buy-in. This quality improvement initiative was determined by the VA Providence Healthcare System Institutional Review Board to not require regulatory review.

### ACORN Program Description

An interprofessional Advisory Board including clinical leadership as well as staff physicians, psychologists, social workers, informaticists, and health services researchers met biweekly over 6 months to develop the ACORN initiative. VA, the country’s largest integrated healthcare system, has been a national leader in the integration of social care into medical care. Our team sought to develop a Veteran-tailored HRSN screening and referral initiative that could be efficiently scaled throughout VA.

Though numerous HRSN screening tools exist, few have been validated and none are specifically designed for Veterans.^10^ We created a tablet-based instrument that could be self-administered through a VA-developed electronic screening platform (eScreening).^11^ Veterans’ responses were then imported by nurses into a clinical note in the electronic health record (EHR) for clinical team review. Our team additionally developed geographically tailored resource guides with VA and non-VA resources to help connect Veterans with needed services.

### Development of the ACORN Screening Tool

We compiled candidate HRSN screening domains from expert bodies including the National Academy of Medicine^1^ and Centers for Medicare & Medicaid Services.^2^ The Advisory Board then determined which HRSNs could be addressed through VA, or other federal, state, or community resources, as our goal was to screen only for needs that could be addressed given existing services. Final ACORN domains included food, housing, utilities, transportation, education, employment, legal needs, interpersonal violence, and social isolation.

After finalizing domains, we reviewed measures from widely used HRSN screening tools.^3,12–15^ To facilitate integration of ACORN into clinical workflows, we used existing VA screening questions for food insecurity^8^ and housing instability.^7^ For the remaining domains, we sourced questions used in other established screeners.^3,^^12,15–19^ For those domains without existing measures, or for which existing measures were not applicable for our population or setting, we adapted or developed new questions based on input from the Advisory Board and other subject matter experts. To ensure acceptability and clarity, we refined the screener based on cognitive testing with 10 men and 8 women Veterans. Veterans were recruited from the VA’s Compensated Work Therapy program^20^ (*n* = 11), which provides vocational rehabilitation services, and 7 Veterans who were also VA employees. The final screening instrument (Appendix) was integrated into eScreening.^11^

### Development of Geographically Tailored Resource Guides

To ensure clinical staff were equipped with information to address Veterans’ self-identified needs and that Veterans left their appointment with relevant resources, we developed geographically tailored resource guides corresponding to each domain in the ACORN screener.^21^ We partnered with VA social workers and community-based organizations to identify high-yield, broadly generalizable resources. Each 1-page guide included points of contact and quick response (QR) codes linked to corresponding program websites. Information for designated VA contacts, often social workers, was also provided on each guide.

### Pre-clinic Integration

Prior to ACORN’s launch, each site identified a nurse champion with whom 2 team members (LER, SC) met weekly for 5 months before the launch. Our team worked alongside the champions and clinical and administrative staff to create site-specific standard operating procedures, encourage staff buy-in, and provide training on ACORN and eScreening. This collaborative approach allowed for tailoring to sites’ unique needs and staffing levels. Both sites identified nurses, who administer other clinical screenings during patient intake, as the most appropriate staff to review screening results with Veterans and provide follow-up support including distribution of resource guides. We also designated specific screening results warranting more intensive follow-up (e.g., urgent food, housing, and utility needs; interpersonal violence; social isolation) and developed workflows to ensure referrals to appropriate VA clinical services. All screening and referral processes were detailed in an implementation guide.

### Clinical Implementation

A convenience sample of Veterans presenting for care at each clinic was asked to complete the ACORN screener in the waiting room. Veterans were handed a tablet by an administrative clerk upon check-in. Positive responses were automatically flagged in eScreening to streamline identification of needs requiring follow-up. For non-urgent needs, nurses provided Veterans with corresponding resource guides. For urgent food, housing, and/or utility needs, interpersonal violence, or social isolation, resource guides were shared and nurses obtained Veteran consent to place a referral or facilitate a warm hand-off to social work and/or mental health.

Clinical implementation started at both sites in October 2019. Once implemented, ACORN team members met weekly with nurse champions to discuss operational concerns and develop any needed workflow modifications. Given the rapid shift to telehealth at the start of the COVID-19 pandemic, our team worked with clinical informaticists to develop an ACORN EHR template that staff could administer during phone and video visits. The suburban clinic pilot ran through May 2020; the urban clinic stopped screening in March 2020 due to pandemic-related staffing limitations.

### Data Collection and Measures

In addition to HRSNs on the ACORN screener, demographic data including sex, age, race, ethnicity, marital status, and VA enrollment priority were obtained from the EHR. Enrollment priority refers to a determination of Veterans’ eligibility for and cost-share associated with VA health benefits, as well as service-connected disability compensation.^22^ We collapsed enrollment priority into 3 categories: Veterans receiving some percentage of service-connected disability compensation, Veterans with no service-connected disability compensation who are low-income, and those with no service-connected disability compensation who are not low-income (above the VA means test).

To understand Veterans’ experiences with ACORN, a purposive sample of Veterans from the urban clinic (*n* = 6) and suburban clinic (*n* = 9) were recruited for brief (~15–20 min) semi-structured telephone interviews 2 weeks after completing ACORN. Sampling was stratified across clinic sites by Veterans with zero identified needs (*n* = 7) or  1 or more needs (*n* = 8), with attempts to oversample women (*n* = 9) and Veterans from racial and ethnic minority groups (*n* = 4; 2 identified as non-Hispanic Black, 1 as multi-racial, and 1 as Hispanic). Ages of Veterans interviewed ranged from 35 to 74 years. Semi-structured interview guides were developed through an iterative process. Domains included Veterans’ reactions to being asked about HRSNs, screening acceptability, attitudes towards continued VA screening, preferences for screening administration, and motivators and barriers to use of resource guides. Interviews were conducted by project staff experienced in qualitative methods, audio-recorded, and transcribed verbatim.

### Analysis

Standard descriptive statistics were calculated for all variables. Chi-square tests were used to assess differences in characteristics by clinic site. Quantitative analyses were conducted using R, version 4.1.2.

Two team members (LER, AJC) independently analyzed interview transcripts using rapid qualitative analysis.^23,24^ Transcripts were summarized in a template based on the interview guide, summaries compared, and discrepancies resolved through consensus. Summaries were then transferred to matrices^25^ and examined by both need burden (zero needs versus 1 or more needs) and clinic site to identify and synthesize key trends.

## RESULTS

### Sample Characteristics

Overall, 268 Veterans were screened: 84 at the urban clinic and 184 at the suburban clinic. Compared with Veterans screened at the suburban clinic, Veterans at the urban clinic were younger (mean age 45 years vs. 61 years, *p* =  < 0.001), and more likely to identify as a racial and/or ethnic minority (16% non-Hispanic Black and 7% Hispanic, vs. 2% non-Hispanic Black and 3% Hispanic, respectively, *p* =  < 0.001). Veterans at the urban clinic were also significantly more likely to be non-married/non-partnered (73% vs. 35%, *p* =  < 0.001), and low-income (16% vs. 11%, *p* = 0.006) (Table 1).

**Table 1:** Characteristics of Veterans Screened

Characteristics	Overall (*n* = 268)	Urban women’s health clinic (*n* = 84)	Suburban community-based clinic (*n* = 184)	*p*-value
Gender, *n* (%)				< 0.001*
Female	96 (35.8)	84 (100.0)	12 (6.5)	
Age, mean (SD)	55.9 (18.6)	45.0 (14.2)	61.0 (18.2)	< 0.001*
Age, *n* (%)				< 0.001*
18–34	49 (18.3)	25 (29.8)	24 (13.0)	
35–49	57 (21.3)	29 (34.5)	28 (15.2)	
50–64	55 (20.5)	23 (27.4)	32 (17.4)	
65–79	83 (31.0)	7 (8.3)	76 (41.3)	
≥ 80	24 (9.0)	0 (0.0)	24 (13.0)	
Race/Ethnicity, *n* (%)				< 0.001*
Non-Hispanic White	219 (81.7)	58 (69.0)	161 (87.5)	
Non-Hispanic Black	16 (6.0)	13 (15.5)	3 (1.6)	
Hispanic	11 (4.1)	6 (7.1)	5 (2.7)	
Other	22 (8.2)	7 (8.3)	15 (8.2)	
Marital status, *n* (%)				< 0.001*
Married/partnered	133 (49.6)	20 (23.8)	113 (61.4)	
Divorced/separated/widowed	55 (20.5)	25 (29.8)	30 (16.3)	
Single, never married	70 (26.1)	36 (42.9)	34 (18.5)	
Missing	10 (3.7)	3 (3.6)	7 (3.8)	
Enrollment priority^a^, *n* (%)				0.006*
SC disability^b^	170 (63.4)	60 (71.4)	110 (59.8)	
Non-SC and low-income^c^	34 (12.7)	13 (15.5)	21 (11.4)	
Non-SC and not low-income^d^	59 (22.0)	8 (9.5)	51 (27.7)	
Missing	5 (1.9)	3 (3.6)	2 (1.1)	
Social need burden				0.001*
0 needs	133 (49.6)	27 (32.1)	106 (57.6)	
1 need	68 (25.4)	26 (31.0)	42 (22.8)	
2 needs	35 (13.1)	16 (19.0)	19 (10.3)	
≥ 3 needs	32 (11.9)	15 (17.9)	17 (9.2)	

^a^Enrollment priority determines US Veterans’ eligibility for and cost-share associated with VA health benefits

^b^Service-connected (SC) disability provides a monetary benefit to Veterans determined by VA to be disabled by an injury or illness that was incurred or aggravated during active military service^22^

^c^Non-service connected Veterans determined by the VA to be low-income

^d^Non-service connected Veterans who have income above the VA administered means test

Chi-square tests; *statistical significance at *p* < .05

Overall, 50% of Veterans screened identified 1 or more HRSN (Table 1). Women were significantly more likely than men to screen positive for at least 1 need (66% vs. 42%, *p* =  < 0.001; Table 2). Though Veterans from racial and ethnic minority groups had higher rates of HRSN than non-Hispanic white Veterans, as did Veterans who were non-married/partnered or low-income, these differences were not significant (Table 2).

**Table 2:** Characteristics of Veterans Screened, by Social Need Burden

Characteristics	0 needs (*n* = 133)	≥ 1 need (*n* = 135)	*p*-value
Gender, *n* (%)			< 0.001*
Female	33 (34.4)	63 (65.6)	
Male	100 (58.1)	72 (41.9)	
Age, mean (SD)	55.6 (19.7)	56.3 (17.5)	0.765
Age, *n* (%)			0.05
18–34	29 (59.2)	20 (40.8)	
35–49	27 (47.4)	30 (52.6)	
50–64	18 (32.7)	37 (67.3)	
65–79	46 (55.4)	37 (44.6)	
≥ 80	13 (54.2)	11 (45.8)	
Race/ethnicity, *n* (%)			0.331
Non-Hispanic White	114 (52.1)	105 (47.9)	
Non-Hispanic Black	5 (31.2)	11 (68.8)	
Hispanic	5 (45.5)	6 (54.5)	
Other	9 (40.9)	13 (59.1)	
Marital status, *n* (%)			0.058
Married/partnered	75 (56.4)	58 (43.6)	
Divorced/separated/widowed	19 (34.5)	36 (65.5)	
Single, never married	34 (48.6)	36 (51.4)	
Missing	5 (50.0)	5 (50.0)	
Enrollment priority^a^, *n* (%)			0.140
SC disability^b^	80 (47.1)	90 (52.9)	
Non-SC and low-income^c^	14 (41.2)	20 (58.8)	
Non-SC and not low-income^d^	35 (59.3)	24 (40.7)	
Missing	4 (80.0)	1 (20.0)	

^a^Enrollment priority determines US Veterans’ eligibility for and cost-share associated with VA health benefits

^b^Service-connected (SC) disability provides a monetary benefit to Veterans determined by VA to be disabled by an injury or illness that was incurred or aggravated during active military service^22^

^c^Non-service connected Veterans determined by the VA to be low-income

^d^Non-service connected Veterans who have income above the VA administered means test

Chi-square tests; *statistical significance at *p* < .05

**Figure 1:**
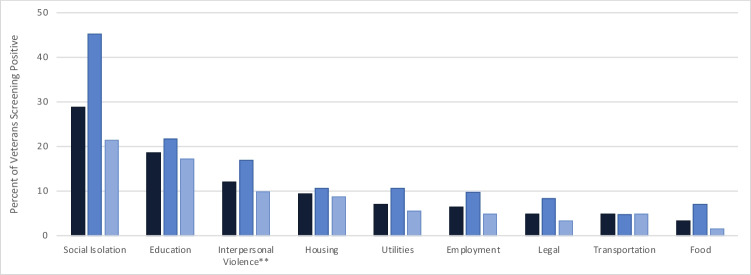
Health-related social needs among Veterans screened, by site. Dark/navy blue bar: overall; medium blue bar: urban women’s health clinic; light blue/gray bar: suburban community-based clinic. *Wording of the interpersonal violence question was modified in March 2020, due to clinic nurses finding high false positive rates when conducting follow-up assessments based on a positive screen. See the Appendix for original and modified wording.

The most commonly reported HRSNs across both clinics were social isolation (29% overall: 45% urban clinic; 21% suburban clinic), educational needs (19% overall: 22% urban clinic; 17% suburban clinic), interpersonal violence (12% overall: 17% urban clinic; 10% suburban clinic), housing instability (9% overall: 11% urban clinic; 9% suburban clinic), and utility concerns (7% overall: 11% urban clinic; 6% suburban clinic) (Fig. 1). Twenty percent of Veterans screened endorsed at least 1 form of material hardship including housing, food, utilities, and/or transportation insecurities (25% urban clinic, 17% suburban clinic, data not shown).

All Veterans interviewed reported comfort with VA screening for HRSNs, that screening for HRSNs was important, and that VA should continue screening. Several Veterans noted that systematic screening could help identify HRSNs among Veterans who might not otherwise feel comfortable raising these needs with their providers, or who may be unaware that VA has resources to address HRSNs. One Veteran explained, “It’s something [VA] need[s] to ask…Because there are Veterans that have [social] needs and really don’t know where to reach out or even know how to ask for that kind of help. Or [they] are embarrassed…If the questions are put to us, sometimes it makes it a little easier to say, ‘yeah I need help with that.’” Another Veteran who did not report any unmet needs reflected, “I thought [screening] was a good idea. I’m sure there is a lot of us Vets that do not have [adequate resources]…[Screening] tells me they [VA] are looking out for me.”

Veterans interviewed resoundingly supported ongoing screening for HRSNs, noting that needs change over time. Several mentioned that Veterans may not feel comfortable disclosing needs when first asked, but in the words of one participant, with regular screening, “perhaps you will get them at the right time where they will reach out when they would never before.” A number of Veterans also felt universal screening for HRSNs would increase awareness about VA services, whether needed for themselves or for Veteran friends and family. One Veteran suggested either less frequent screening for Veterans who do not endorse any needs on an initial screen or providing Veterans previously screening negative the ability to opt out of rescreening if their circumstances were unchanged.

Participants liked being able to complete the ACORN screener in the waiting room, with one Veteran remarking, “It’s a lot better than looking at a pamphlet wall.” eScreening was described as convenient, fast, and easy to use. While one Veteran noted that screening might feel more personal if verbally administered by staff, several Veterans appreciated the privacy afforded by the tablet and “feel[ing] like you can answer without being judged.”

The majority of Veterans interviewed who received resource guides based on identified needs reported finding the content well laid out and easy to understand, although none reported using the resource guides to connect with services by the 2-week follow-up. Reasons for not using resource guides included not yet having had the time to connect with services, not having an immediate need, and in some cases not remembering receiving the guides. Several Veterans, however, reported planning to use the resource guides in the near future.

## DISCUSSION

In this Veteran-tailored HRSN screening and referral initiative, half of Veterans screened reported  1 or more HRSNs (42% in a suburban clinic; 68% in an urban clinic). Across both clinics, social isolation was the greatest unmet need, which was endorsed by nearly one-third of Veterans. One in 5 participants reported at least some form of material hardship. Regardless of whether or not they reported HRSNs, Veterans felt screening was acceptable and important, and that VA should continue screening.

To our knowledge, this is the first study of a systematic HRSN screening and referral program among Veterans. The high rate of social isolation among those screened is particularly notable, as our data were collected primarily before the COVID-19 pandemic and reported social isolation and loneliness—which are associated with numerous adverse health outcomes among Veterans including increased risk for re-hospitalization and decreased well-being^26,27^—have worsened globally since that time.^28,29^ We also found substantially higher rates of food insecurity and housing instability than has been previously reported within VA despite our using the same screening questions.^7,30,31^ These findings may be due, in part, to the fact that ACORN was Veteran self-administered whereas VA clinical reminders are staff-administered. Prior research has found patients prefer self-administered over staff-administered screening for HRSNs^32–34^, and that disclosure rates for food or financial insecurity and other sensitive topics such as exposure to violence and substance use are higher with self-administered screening.^32,34–36^ Rates of other HRSNs reported in ACORN were also higher than those found in other studies using VA EHR data such as ICD-10 codes^37^, which are known to be underutilized.^38–40^

The ability to systematically screen for HRSNs and allow patients to self-administer screening is a strength of ACORN and has the potential to markedly improve the quality and accuracy of VA data. Self-administered, tablet-based screening allows for increased privacy, greater efficiency, and real-time review of responses by clinical staff.^36,41^ Previous research has further demonstrated that Veterans prefer tablet-based to paper-based screening, and that completion rates were significantly higher with tablet-based screening.^11^ Future work is needed to explore potential differences in Veteran and staff acceptability, perceived feasibility, and HRSN disclosure rates between Veteran self-administered and staff-administered screening.

Our findings of high rates of acceptability of screening for HRSNs are consistent with prior work in non-VA healthcare settings.^42–44^ Notably, in our study and others, even patients who did not endorse unmet needs spoke to the importance of universal screening. Future work is needed to establish an evidence base for optimal screening frequency, and how frequency may differ for routine screening versus follow-up assessments once needs are identified or in high-risk populations where needs may be more prevalent.

Despite widespread support for healthcare-based social needs screening, previous work has found many patients screening positive for HRSNs decline assistance or do not follow-up with offered resources.^42,43,45–54^ Among the subset of Veterans we interviewed, none who screened positive for HRSNs had used resource guides at 2-week follow-up, although several noted planning to use them in the future. Consistent with prior work, reasons Veterans did not use resources included not feeling they had a current need, competing priorities, and not remembering receiving the guides.^42,45,46,49,52^ Since this pilot, ACORN has expanded to over 15 VA facilities nationally across different clinical settings, and in collaboration with our operational partners, we have worked with local teams to support the development of site-specific workflows to ensure appropriate follow-up and referrals are provided.^55^ We have also established a Community of Practice to support and sustain implementation at both new and existing sites. Recognizing that optimal practices may vary based on patient preferences and needs, local resources, and staffing, there is likely not a one-size-fits-all approach to identifying and addressing HRSNs. Additional research is needed to better understand which patients may benefit from “low-touch” interventions such as tailored resource guides, and which patients need “higher-touch” interventions such as navigation or case management.

Our findings should be interpreted within the context of several limitations. First, this initiative took place in 2 VA primary care clinics in New England, which may limit generalizability. Second, COVID-19 lockdowns led to a drop in screenings in the suburban clinic due to decreases in clinic volume and staffing, and screening in the urban clinic halted completely in March 2020. Future work should examine whether enabling Veterans to securely complete an electronic screener remotely, such as through a patient portal, may help scale HRSN screening efforts and response. However, it is important to note that digital health inequities (i.e., lack of access to an internet-connected device and/or low digital health literacy) may prevent some Veterans from accessing these tools. Third, numbers and demographics of Veterans declining screening were not reliably collected by clinic staff and findings may have been susceptible to selection bias. Demographics of those screened, however, were generally similar to the broader patient population in each of the clinics with the exception that our sample was overall younger and more likely to be single. Fourth, Veteran-reported acceptability of ACORN was based on interviews with a subset of participants and may not be representative of all patients. We did, however, purposively sample Veterans who did and did not screen positive for HRSNs, and reached saturation within each stratum. Lastly, reported acceptability and importance of screening may have been subject to social desirability bias, although we tried to mitigate this bias by ensuring all respondents knew their responses would be anonymized and asking opened-ended questions in a non-judgmental way. Despite these limitations, our findings have important implications for our understanding of identifying and addressing HRSNs in healthcare settings.

## CONCLUSION

A self-administered electronic HRSN screening and referral program identified that Veterans have substantial HRSNs and want VA to screen for and address these needs. Although health systems cannot independently address upstream determinants of health, they can serve as leaders and advocates in the identification and management of patients’ HRSNs. Screening for HRSNs is a critical step towards connecting patients with needed services, identifying gaps in the current service delivery system, and informing future resource allocation.

## Supplementary Information

Below is the link to the electronic supplementary material.

Supplementary file1 (PDF 250 KB)
