# Supplementary material for: Implementing a Social Needs Screening and Referral Program Among Veterans: Assessing Circumstances & Offering Resources for Needs (ACORN)
Source: J Gen Intern Med. 2023 May 10;38(13):2906–13. doi: 10.1007/s11606-023-08181-9 (PMC10171907; doi:10.1007/s11606-023-08181-9)
Supplement: Supplementary file 1 — Supplementary file1 (PDF 250 KB) [file 11606_2023_8181_MOESM1_ESM.pdf]

APPENDIX A: ACORN Screening Tool and Source Table

These questions are designed to identify any unmet need(s) you might have, so that the VA can follow up with the appropriate resources and support. Please answer each question to the best of your ability; however, you may skip questions if you are unsure of the answer or would prefer not to respond.

(1) In the past three months, did you ever run out of food and you were not able to access more food or have the money to buy more food?

☐ Yes ☐ No

(2) Do you currently have any concerns about having enough food?

☐ Yes ☐ No

(3) Do you need help getting food for this week?

☐ Yes ☐ No

(4) In the past two months, have you been living in stable housing that you own, rent, or stay in as part of a household?

☐ Yes ☐ No

(5) Are you worried or concerned that in the next two months that you may NOT have stable housing that you own, rent, or stay in as part of a household?

☐ Yes ☐ No

(6) Are you currently without a place to stay?

☐ Yes ☐ No

(7) Do you have trouble paying for your utilities (i.e., gas, electricity, phone, and water)?

☐ Yes ☐ No

(8) Has the electric, gas, oil, or water company threatened to shut off services in your home?

☐ Yes ☐ No ☐ Already shut off

(9) Has lack of transportation kept you from medical appointments, meetings, work, or from getting things needed for daily living?

☐ Yes ☐ No

(10) Are you currently dealing with any legal issues, for which you may need assistance?

☐ Yes ☐ No

(11) Please select the answer that most represents your current experience. How often do you feel lonely or isolated from those around you?

☐ Never ☐ Rarely ☐ Sometimes ☐ Often ☐ Always

(12) Please select the answer that most represents your current experience. How often does anyone, including family and friends, mentally, physically, sexually, and/or verbally hurt you?[1]

☐ Never ☐ Rarely ☐ Sometimes ☐ Often ☐ Always

(13) Do you want help finding or keeping work or a job?

☐ Yes, help finding work ☐ Yes, help keeping work ☐ I do not need or want help

(14) Do you want more information about educational benefits and resources for Veterans?

☐ Yes ☐ No

[1]Wording of the interpersonal violence question was modified in March 2020, due to clinic nurses finding high false positive rates when conducting follow-up assessments based on a positive screen. Revised wording: "Please select the answer the most represents your current experience. How often does anyone close to you physically hurt you or threaten you with harm?"

Source Table for HRSN Items Used in the ACORN Screening Tool.

When derived from the Centers for Medicare and Medicaid Services (CMS) Accountable Health Communities (AHC) Screener, the original source is cited per AHC guidance. Questions without citations were developed by the U.S. Department of Veterans Affairs (VA) ACORN core team and collaborators across multiple VA offices and sites.

Note: This is the source table is for the original ACORN Screener, which has since been revised. The most updated version can be found at: https://www.va.gov/HEALTHEQUITY/docs/ACORN_Screening_Tool.pdf

Domain	Question	Original Source	Modifications	Citation
**Housing**	In the past two months, have you been living in stable housing that you own, rent, or stay in as part of a household?<br>• Yes<br>• No	VA Clinical Reminder	None	VA National Center on Homelessness Among Veterans. "Homeless Screener." U.S. Department of Veterans Affairs, September 2020. https://www.va.gov/HOMELESS/nchav/resources/prevention/homeless-screener.asp
	Are you worried or concerned that in the next two months you may NOT have stable housing that you own, rent, or stay in as part of a household?<br>• Yes<br>• No			
	Are you currently without a place to stay?<br>• Yes<br>• No	Developed by VA ACORN team	n/a (new item)	Developed by VA ACORN team
**Food**	In the past three months, did you ever run out of food and you were not able to access more food or have the money to buy more food?<br>• Yes<br>• No	VA Clinical Reminder	None	Veterans Health Administration Food Insecurity Clinical Reminder, 2017-2021.
	Do you currently have any concerns about having enough food?<br>• Yes<br>• No	Developed by VA ACORN team	n/a (new item)	Developed by VA ACORN team

Domain	Question	Original Source	Modifications	Citation
	Do you need help getting food for this week?<br>• Yes<br>• No	Developed by VA ACORN team	n/a (new item)	Developed by VA ACORN team
**Utilities**	Do you have trouble paying for your utilities (i.e., gas, electricity, phone, and water)?<br>• Yes<br>• No	WellRx	Expanded the original list of utilities included in the WellRx question to include difficulty paying for water	Adapted from Page-Reeves J, Kaufman W, Bleecker M, Norris J, McCalmont K, Ianakieva V, Ianakieva D, Kaufman A. Addressing Social Determinants of Health in a Clinic Setting: The WellRx Pilot in Albuquerque, New Mexico. J Am Board Fam Med. 2016 May-Jun;29(3):414-8.
	Has the electric, gas, oil, or water company threatened to shut off services in your home?<br>• Yes<br>• No<br>• Already shut off	AHC Screener (modified from Children's Health Watch – Household Energy Security Screener	Omitted "in the past 12 months" from the AHC question	Adapted from Cook, J. T., Frank, D. A., Casey, P. H., Rose-Jacobs, R., Black, M. M., Chilton, M., . . . Cutts, D. B. (2008). A Brief Indicator of Household Energy Security: Associations with Food Security, Child Health, and Child Development in US Infants and Toddlers. Pediatrics, 122(4), 867-875.
**Trans-portation**	Has lack of transportation kept you from medical appointments, meetings, work, or from getting things needed for daily living?<br>• Yes<br>• No	PRAPARE	Condensed response options to yes/no in place of longer list provided in PRAPARE. We also excluded PRAPARE's decline to answer response, since we explicitly provided the following directions to respondents, "Please answer each question to the best of your ability; however, you may skip questions if you are unsure of the answer or would prefer not to respond."	Adapted from National Association of Community Health Centers and Partners, National Association of Community Health Centers, Association of Asian Pacific Community Health Organizations, Association OPC, Institute for Alternative Futures. (2017). PRAPARE. http://www.nachc.org/research-and-data/prapare/

Domain	Question	Original Source	Modifications	Citation
**Legal**	Are you currently dealing with any legal issues, for which you may need assistance?<br>• Yes<br>• No	Developed by VA ACORN team	n/a (new item)	Developed by VA ACORN team
**Social Isolation and Loneliness**	Please select the answer that most represents your current experience. How often do you feel lonely or isolated from those around you?<br>• Always<br>• Often<br>• Sometimes<br>• Rarely<br>• Never	AHC Screener (modified from AARP Survey)	Added to AHC question "Please select the answer that most represents your current experience"	Adapted from Anderson, G. Oscar and Colette E. Thayer. Loneliness and Social Connections: A National Survey of Adults 45 and Older. Washington, DC: AARP Research, September 2018. https://doi.org/10.26419/res.00246.001
**Inter-personal Safety**	Please select the answer that most represents your current experience. How often does anyone, including family and friends, mentally, physically, sexually, and/or verbally hurt you?<br>• Always<br>• Often<br>• Sometimes<br>• Rarely<br>• Never<br><br>* In March 2020, question modified to "Please select the answer that most represents your current experience. How often does anyone close to you physically hurt you or threaten you with harm?"<br>• Always<br>• Often<br>• Sometimes<br>• Rarely<br>• Never	AHC screener (modified from Hurt, Insult, Threaten, and Scream (HITS) Tool for Intimate Partner Violence Screening to broaden to interpersonal violence	Condensed 4 item measure to a single question	Adapted from Sherin, K. M., Sinacore, J. M., Li, X. Q., Zitter, R. E., & Shakil, A. (1998). HITS: a Short Domestic Violence Screening Tool for Use in a Family Practice Setting. Family Medicine, 30(7), 508-512.

Domain	Question	Original Source	Modifications	Citation
**Employ-ment**	Do you want help finding or keeping work or a job?<br>• Yes, help finding work<br>• Yes, help keeping work<br>• I do not need or want help	Centers for Medicare & Medicaid Services - Accountable Health Communities Technical Expert Panel	None	Identifying and Recommending Screening Questions for the Accountable Health Communities Model (2016, July) Technical Expert Panel discussion conducted at the U.S. Department of Health and Human Services, Centers for Medicare & Medicaid Services, Baltimore, MD.
**Education**	Do you want more information about educational benefits and resources for Veterans?<br>• Yes<br>• No	Developed by VA ACORN team	n/a (new item)	Developed by VA ACORN team
